# The Development of Horner Syndrome following a Stabbing

**DOI:** 10.1155/2014/461787

**Published:** 2014-10-07

**Authors:** Muhammet Sayan, Ali Çelik

**Affiliations:** ^1^Department of Thoracic Surgery, Aksaray State Hospital, 68200 Aksaray, Turkey; ^2^Department of Thoracic Surgery, Gazi University School of Medicine, Besevler, 06500 Ankara, Turkey

## Abstract

The features of Horner Syndrome are miosis, ptosis, enophthalmos, and anhidrosis on the same side as the etiologic pathology. Its causes include tumours, aneurysms, neck and chest surgery, and neck and chest trauma. This paper presents a case of Horner Syndrome due to a haemopneumothorax following penetrating chest trauma.

## 1. Introduction

Horner Syndrome, or oculosympathoparesis, first described by Johann Friedrich Horner in 1929, classically presents with ipsilateral ptosis, miosis, and anhidrosis [1, 2]. It results from an interruption of the oculosympathetic pathway. The causes include tumour infiltration, compression by a lesion such as an aneurysm, iatrogenic causes, and traumatic injuries [1]. Other very rare etiologic factors, such as highly positioned chest tubes and severe blunt neck and chest trauma, are reported in the literature [3].

## 2. Case Report

**Figure 1:**
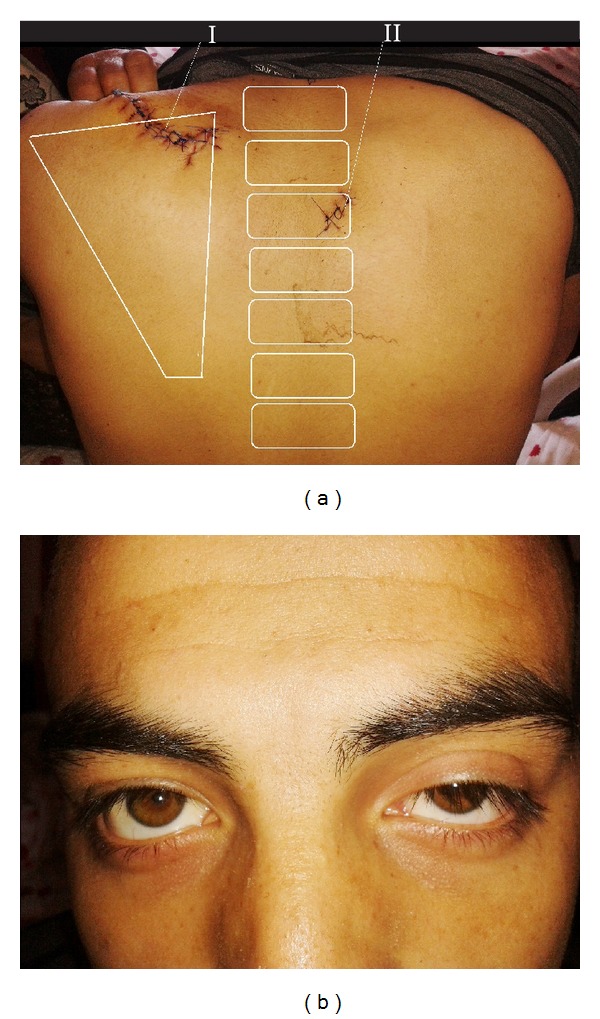
(a) (I) First stab wound that deepened at medial region (sutured). (II) Other superficial wound. (b) Left miosis, ptosis, and enophthalmos.

An 18-year-old male patient presented to emergency medicine after suffering a stab wound to the left-upper posterior hemithorax and paravertebral region (Figure 1(a)). Physical examination revealed left-sided miosis and ptosis, subcutaneous emphysema of the neck and left upper hemithorax, and decreased breath sounds on the left hemithorax (Figure 1(b)). Computerized tomography of the chest was performed and a large, left-sided pneumothorax and minimal haemothorax were detected.

Cranial and vertebral MRI was performed in order to rule out brain and spinal cord damage and no abnormalities were found. A chest tube was inserted in the left hemithorax at the sixth intercostal space under local anaesthesia and closed underwater seal drainage was performed. The patient was evaluated by specialists in neurology and neurosurgery regarding the features of Horner Syndrome, but no additional treatment was recommended. On postoperative day three, lung expansion was achieved and the chest tube was removed. The patient was discharged on the fourth day following the injury, although the features of Horner Syndrome had not resolved. The patient was followed up for 1 year but improvement was not seen.

## 3. Discussion

Horner Syndrome results from an interruption of the sympathetic innervation to the eye and ocular adnexae. The sympathetic innervation of the eye occurs in a three-order system. The first-order neuron arises from the hypothalamus. The second-order neuron connects the stellate and middle cervical ganglia and terminates in the superior cervical ganglia. The third-order neuron exits from the superior cervical ganglion and innervates the m.levator palpebrae superior and m.dilator papillae [1]. This syndrome consists of miosis, ptosis, visible enophthalmos, and anhidrosis ipsilateral to the lesion [4].

Horner Syndrome is caused by an interruption of the oculosympathetic pathway at any point between its origin in the hypothalamus and the eye. The causes of this interruption include tumour infiltration, compression by an aneurysm, iatrogenic causes, and traumatic injury [1]. Iatrogenic causes include central venous access, thyroidectomy, sympathetic ganglion blockade, carotid angiography, thoracic sympathectomy, highly positioned chest tubes, and various surgeries of the neck [5, 6]. Traumatic causes of Horner Syndrome include penetrating trauma, such as a stab or bullet wound, and blunt trauma of the neck and upper thorax. In terms of neoplasms, there are benign (*schwannoma*,* carotid body tumor thyroid adenoma, etc.*) and malignant (*thyroid cancer, Hodgkin lymphoma, lung cancer, sarcoma, and metastasis*) causes. Other very rare etiologies are birth trauma, carotid artery dissection, Giant cell arteritis, Wegener granulomatosis, Herpes zoster, and migraine headache [1].

Topical pharmacologic agents such as cocaine or phenylephrine can be used to confirm a diagnosis of Horner Syndrome [2]. Cocaine acts by blocking reuptake of norepinephrine in the neuromuscular junction of the iris dilator muscle, causing the pupil in a normal eye to dilate, while incomplete dilation is seen in Horner Syndrome.

When Horner Syndrome is caused by compression of the oculosympathetic pathway, as occurs with tumours and aneurysms, the cause of the compression should be treated. However, as in our case, there is no definitive treatment for a direct cut in the pathway of the nerve.
